# Intestinal-Type Villous Adenoma of the Urinary Bladder Presenting as an Asymptomatic Diffuse Plaque-Like Trigonal Lesion: A Rare Case With Multimodal Clinicopathologic Correlation

**DOI:** 10.7759/cureus.116348

**Published:** 2026-09-16

**Authors:** Sushmitha Kothapalli, Thalapathi Raja, Pavithra V, Sandhya Sundaram, Sriram Krishnamoorthy, Roshan Yedulla Reddy

**Affiliations:** 1 Urology, Sri Ramachandra Institute of Higher Education and Research, Chennai, IND; 2 Pathology, Sri Ramachandra Institute of Higher Education and Research, Chennai, IND

**Keywords:** cystitis glandularis, intestinal metaplasia, transurethral resection, urinary bladder, villous adenoma

## Abstract

Villous adenoma of the urinary bladder is a rare, benign glandular neoplasm with intestinal differentiation. This neoplasm is frequently associated with chronic urinary irritation and intestinal metaplasia of the urothelium. Fewer than 50 cases have been documented, and limited information is available regarding its clinicoradiologic spectrum.

A 51-year-old man with severe anemia was found to have multiple broad-based exophytic lesions of the bladder on ultrasound. There was no hematuria, mucosuria, or other lower urinary tract symptoms. A minimally enhancing, plaque-like lesion measuring approximately 5 × 1 cm was observed in the trigone and extended to both vesicoureteric junctions. CT urography showed no obstruction or invasion by the lesion. Cystoscopy revealed a velvety, villiform lesion involving the bladder base, and complete transurethral resection was performed. Histopathological examination showed an intestinal-type villous adenoma composed of papillary and villous fronds lined by pseudostratified columnar epithelium with goblet cells, arising in a background of extensive cystitis cystica et glandularis with intestinal metaplasia and focal high-grade dysplasia. No invasive carcinoma was identified. Follow-up cystoscopy at six months demonstrated no recurrence.

To the best of our knowledge, this represents the first reported case of an entirely asymptomatic intestinal-type villous adenoma presenting as a diffuse, plaque-like trigonal lesion extending across both vesicoureteric junctions. This case provides further support for the hypothesis of a bladder carcinogenesis pathway involving an irritation-metaplasia-adenoma sequence. Although these lesions have benign histological appearances, they warrant complete histological evaluation and follow-up because of their documented predisposition to developing synchronous or metachronous malignant lesions. This case emphasizes the importance of complete histological evaluation and continued follow-up.

## Introduction

Villous adenoma of the urinary bladder is an exceptionally rare benign glandular neoplasm characterized by intestinal-type epithelial differentiation and histomorphologic resemblance to colorectal villous adenomas [1]. Although relatively common in the intestines, its occurrence in the urinary bladder is remarkably infrequent, with fewer than 50 documented cases reported in the scientific literature [2]. This neoplasm predominantly affects older males and usually arises in areas where metaplastic transformation is likely to occur, such as the bladder trigone, bladder neck, and dome of the urinary bladder [1].

Bladder villous adenomas are clinically characterized by painless hematuria, irritative lower urinary tract symptoms, or mucosuria due to mucin excretion [1,3]. In contrast, lesions discovered incidentally in asymptomatic patients are very uncommon and can pose diagnostic challenges [1,3]. Radiologic findings in these cases lack distinctive characteristics and can be difficult to distinguish from those of other benign or malignant papillary bladder tumors [1,3]. Consequently, definitive diagnosis relies on pathological examination [1,3]. Histologically, these lesions typically demonstrate villous or papillary projections lined by pseudostratified columnar epithelium containing goblet cells [1,3].

Bladder villous adenomas have clinical significance beyond their rarity. Increasing evidence suggests that they develop in the setting of chronic inflammation and glandular metaplasia of the urothelium, particularly intestinal-type metaplasia associated with cystitis cystica et glandularis [1,2]. This phenomenon provides further support for the proposed "irritation-metaplasia-adenoma-carcinoma" pathway of urinary bladder carcinogenesis, analogous to the "adenoma-carcinoma" sequence described in colorectal cancer [1,2]. Chronic irritation caused by urinary tract infections, bladder obstruction, calculi, or inflammation has been associated with glandular metaplasia of the urothelium [2]. An alternative embryologic hypothesis proposes that these lesions originate from persistent cloacal or urachal remnants capable of intestinal differentiation [2].

However, because of their potential for malignant transformation, villous adenomas are clinically important because of their association with concurrent or subsequent development of malignancy [1,4]. Several documented cases have described these tumors occurring concurrently with adenocarcinoma or urothelial carcinoma, and some have been associated with mucinous adenocarcinoma of the urachus [1,5]. These observations suggest that villous adenomas, particularly those arising in metaplastic urothelium, may harbor malignant potential and therefore require accurate recognition and appropriate management to avoid both underdiagnosis and overtreatment [1,4,5].

Based on the existing evidence, this represents the first documented case of an asymptomatic intestinal-type villous adenoma presenting as a diffuse trigonal plaque lesion involving both vesicoureteric junctions. This report illustrates a unique clinical and radiological presentation, along with excellent radiographic, endoscopic, and histopathologic correlation, emphasizing the proposed irritation-metaplasia-adenoma pathway in bladder carcinogenesis [1,2].

## Case presentation

A 51-year-old man undergoing evaluation for severe anemia was incidentally found to have a suspected urinary bladder lesion on abdominal ultrasonography and was subsequently referred to our tertiary care center for urological evaluation. The patient denied hematuria, mucosuria, dysuria, lower urinary tract symptoms, flank pain, or recurrent urinary tract infections. There was no prior history of bladder calculi, chronic catheterization, schistosomiasis, or previous urological intervention. Physical examination was unremarkable except for pallor. Initial laboratory investigations in August 2025 revealed severe anemia (hemoglobin, 4.9 g/dL), leukopenia (2,970 cells/mm³), and thrombocytopenia (0.74 lakh/mm³). The anemia was markedly macrocytic (MCV, 134.7 fL). Peripheral smear examination demonstrated a dimorphic red-cell population comprising predominantly macro-ovalocytes, along with microcytes and a few microcytic hypochromic cells, with associated leukopenia and thrombocytopenia. Further hematological evaluation showed low serum iron (19 µg/dL), elevated lactate dehydrogenase (1,626 U/L), and a serum vitamin B12 level of 321 pg/mL. There was no history of hematuria or other clinically evident urinary blood loss; therefore, the hematological abnormalities were considered unrelated to the bladder lesion. During the preceding General Medicine admission, the patient received four units of packed red blood cells for correction of severe anemia, along with iron and parenteral vitamin B12 supplementation. At admission to the urology service in September 2025, the hemoglobin level was 10.0 g/dL, the total leukocyte count was 8,120 cells/mm³, and the platelet count was 3.07 lakh/mm³. Serial laboratory values are summarized in Table 1.

**Table 1:** Serial laboratory investigations at initial evaluation and subsequent urology admission. During the intervening General Medicine admission, the patient received packed red blood cell transfusion along with iron and parenteral vitamin B12 supplementation. Reference intervals are those stated in the institutional laboratory reports. RBC: red blood cell, PCV: packed cell volume, MCV: mean corpuscular volume, MCH: mean corpuscular hemoglobin, MCHC: mean corpuscular hemoglobin concentration.

Parameter	Initial evaluation	Urology admission	Institutional reference range
Hemoglobin (g/dL)	4.9	10	13.0-17.0
Total leukocyte count (cells/cumm)	2,970	8,120	4,000-11,000
Neutrophils (POLY) (%)	46.8	75.2	45-70
Immature granulocytes (IG) (%)	2	0.4	<1.0
Lymphocytes (%)	41.1	18.6	25-40
Eosinophils (%)	1	1.5	1-6
Monocytes (%)	9.1	3.6	2-10
Basophils (%)	0	0.7	0-1
RBC count (million/cumm)	1.24	3.31	4.5-5.5
Platelet count (lakh/cumm)	0.74	3.07	1.5-4.5
PCV (%)	16.7	33.9	40-50
MCV (fL)	134.7	122	83-101
MCH (pg)	39.5	38	27-33
MCHC (g/dL)	29.3	33.8	31.5-34.5
Blood urea nitrogen (BUN) (mg/dL)	6	7	6.0-20.0
Creatinine (mg/dL)	0.7	0.7	0.7-1.2
Sodium (mmol/L)	139	142	136-145
Potassium (mmol/L)	4.1	3.9	3.5-5.1
Chloride (mmol/L)	106	108	98-107
Bicarbonate (mmol/L)	21	22	22-29

Ultrasonography of the kidney-ureter-bladder region revealed multiple broad-based, isoechoic, exophytic lesions arising from the posterior bladder wall near the trigone, with the largest measuring approximately 1.0 × 0.5 cm. No internal vascularity was identified on Doppler imaging, and there was no evidence of hydronephrosis or upper urinary tract pathology. Subsequent contrast-enhanced computed tomography (CECT) urography demonstrated an ill-defined, minimally enhancing, plaque-like soft-tissue thickening measuring approximately 5 × 1 cm along the bladder base and trigone, extending across both vesicoureteric junctions.

The lesion exhibited minimal enhancement without evidence of calcification, hemorrhage, extravesical extension, or pelvic lymphadenopathy. Although the lesion extended across both vesicoureteric junctions and caused mild narrowing of the distal ureters, there was no upstream hydroureteronephrosis or delayed contrast excretion on either side. Collectively, the imaging findings suggested an atypical, noninvasive glandular bladder lesion (Figure 1a-1c). Cystoscopic examination revealed a diffuse, velvety, pale-whitish, plaque-like lesion involving the trigone and bladder base, characterized by broad-based villiform and papillary fronds with a mucinous, cauliflower-like appearance. Both ureteric orifices were visualized adjacent to the lesion without evidence of obstruction (Figure 1d). Complete transurethral resection of the bladder tumor (TURBT) was performed, including deep muscle sampling to exclude invasion.

**Figure 1:**
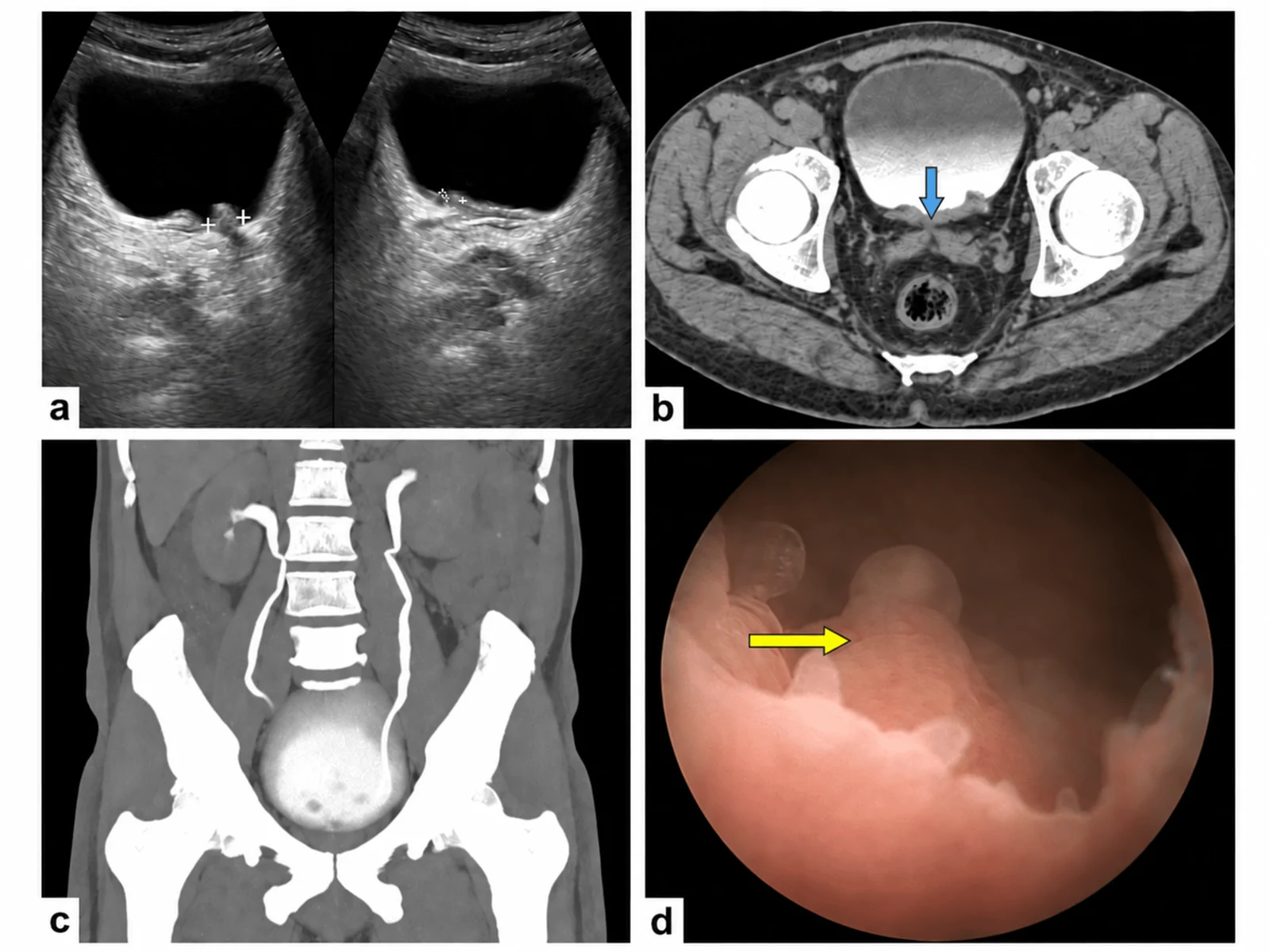
Radiologic and endoscopic appearance of intestinal-type villous adenoma of the urinary bladder. (a) Transabdominal ultrasonography demonstrating broad-based exophytic lesions arising from the bladder base near the trigone. (b) Axial contrast-enhanced CT urography image showing eccentric plaque-like soft-tissue thickening at the bladder base (blue arrow), without evidence of extravesical invasion. (c) Coronal CT urography image demonstrating opacification of both ureters without upstream hydroureteronephrosis, despite involvement of the trigone and both vesicoureteric junctions. (d) Cystoscopic view revealing a pale-whitish, broad-based villiform lesion with lobulated papillary fronds and a cauliflower-like mucinous appearance arising from the trigonal mucosa (yellow arrow).

Histopathological examination demonstrated papillary and villous structures lined by pseudostratified, tall columnar epithelium with focal goblet cells, resembling intestinal mucosa. The lesion showed mild nuclear pseudostratification with focal high-grade dysplasia, without stromal invasion or significant mitotic activity. The background urothelium revealed extensive cystitis cystica et glandularis with intestinal metaplasia merging with the adenomatous epithelium (Figure 2a, 2b). Extensive sampling showed no evidence of adenocarcinoma, urothelial carcinoma, or carcinoma in situ. The final diagnosis was intestinal-type villous adenoma of the urinary bladder arising in a background of cystitis cystica et glandularis.

**Figure 2:**
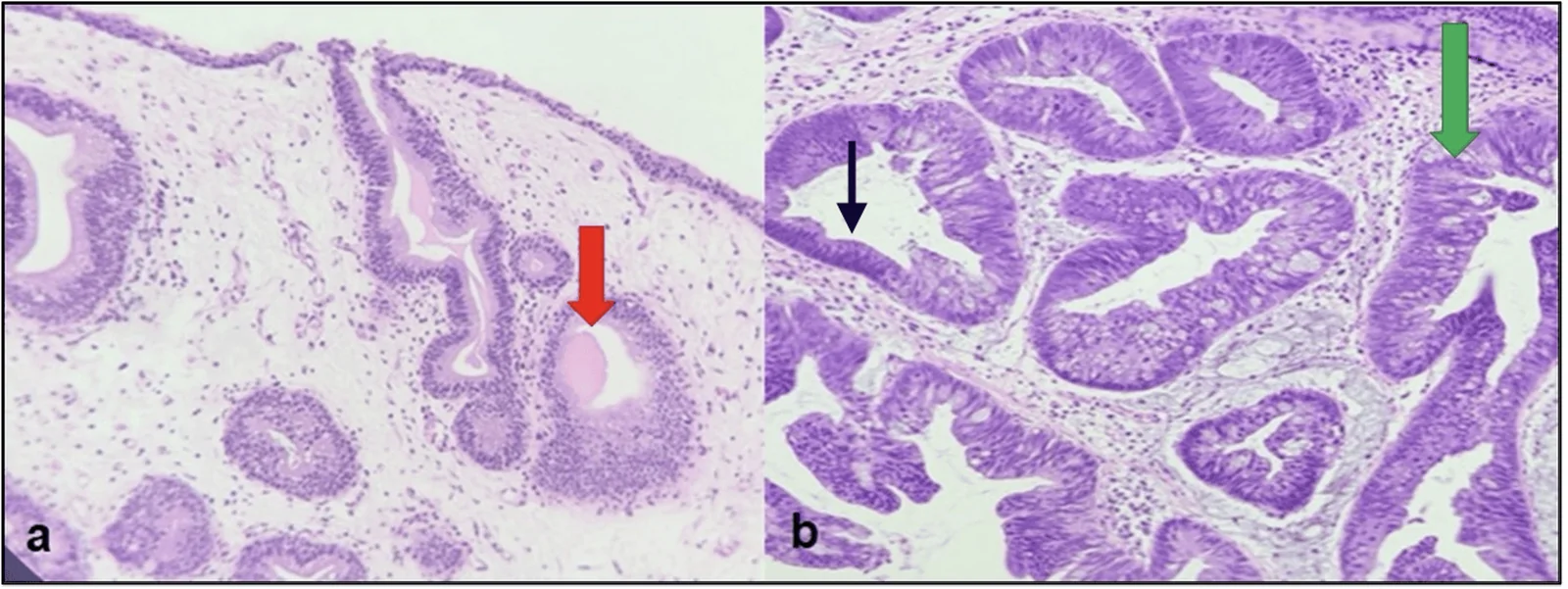
Histopathological features of intestinal-type villous adenoma of the urinary bladder. (a) Low-power hematoxylin and eosin (H&E) photomicrograph (40×) demonstrating intestinal-type villoglandular proliferation with cystically dilated mucin-filled glands (red arrow) within an inflamed lamina propria, without evidence of stromal invasion. (b) High-power H&E photomicrograph (100×) showing complex villiform and glandular architecture lined by tall pseudostratified columnar epithelium with focal nuclear crowding and goblet cell differentiation (green arrow), areas of focal high-grade dysplasia (blue arrow) consistent with intestinal-type villous adenoma. No invasive component is identified.

The postoperative course was uneventful. Follow-up cystoscopic evaluations at three and six months demonstrated no evidence of recurrence, and the patient remains under periodic endoscopic surveillance given the associated field change of intestinal metaplasia and the recognized risk of coexistent malignancy.

## Discussion

Villous adenoma of the urinary bladder is an exceptionally rare glandular neoplasm with intestinal differentiation and remains a diagnostic challenge because of its nonspecific clinical and radiologic presentation [6,7]. Most reported cases occur in older men and typically present with painless hematuria, irritative lower urinary tract symptoms, or mucosuria [1,6]. In contrast, the present case involved a completely asymptomatic patient, with the lesion discovered incidentally during evaluation for anemia. Furthermore, the neoplasm exhibited a rare, diffuse, plaque-like pattern involving the trigone rather than the more common polypoid appearance [1,6]. To the best of our knowledge, such a clinicoradiologic presentation has not been previously described [6,7].

Studies have consistently demonstrated a close relationship between villous adenoma and chronic glandular metaplasia of the urothelium, including cystitis glandularis and cystitis cystica [7-9]. Evidence from multiple sources supports the presence of intestinal metaplasia adjacent to villous adenoma in the context of the irritation-metaplasia-adenoma-carcinoma sequence proposed for urinary bladder carcinogenesis [7-9]. Chronic inflammation, recurrent urinary tract infections, bladder outlet obstruction, calculi, and long-term catheterization have all been implicated as contributing factors [7-9].

Histopathologically, bladder villous adenomas closely resemble their colorectal counterparts and are characterized by villiform or papillary fronds lined by pseudostratified columnar epithelium containing goblet cells [1,9]. The principal differential diagnoses include primary bladder adenocarcinoma, metastatic colorectal adenocarcinoma, florid cystitis glandularis, and urothelial carcinoma with glandular differentiation [1,9]. The absence of stromal invasion and destructive growth favors a diagnosis of benign villous adenoma, although dysplasia may be present [1,9]. In challenging cases, immunohistochemistry may be considered to aid differentiation; villous adenomas typically express intestinal markers such as CK20 and CDX2 but lack expression of urothelial markers such as GATA3 [9,10].

Although pure villous adenomas generally exhibit benign clinical behavior, their biological significance arises from their association with urothelial carcinoma or adenocarcinoma [1,11]. Channer et al. reported a villous adenoma of the urinary bladder associated with intestinal-type cystitis glandularis [12]. In the 18-case series by Seibel et al., 6 cases (33%) were pure villous adenoma, whereas the remaining 12 cases had associated in situ and/or infiltrating adenocarcinoma and/or urothelial carcinoma [13]. Further examples of these associations [14-16] are presented in Table 2. In view of this association, thorough transurethral resection with adequate deep sampling is essential to rule out invasion [6,11].

**Table 2:** Summary of previously published reports of villous adenoma of the urinary tract and their clinicopathological features. TURBT: transurethral resection of bladder tumor, BCG: Bacillus Calmette-Guérin.

Study	Clinical presentation	Site/key findings	Associated pathology	Management/outcome
Channer et al. (1993) [12]	Not specified in the accessible abstract	Villous adenoma of the urinary bladder	Associated intestinal-type cystitis glandularis	Not specified in the accessible abstract
Seibel et al. (2002) [13]	18 patients; age 53-93 years; 67% male	18 cases involving the bladder, urachus, and prostatic urethra	6 pure villous adenomas; 12 with associated in situ and/or infiltrating adenocarcinoma and/or urothelial carcinoma	Outcome available in 8 cases; non-invasive lesions had favorable outcomes, whereas infiltrating carcinoma was associated with metastasis/progression
Sung et al. (2008) [14]	Gross hematuria	Large bladder villous adenoma	No associated malignancy	Complete TURBT; no recurrence
Kato et al. (2013) [15]	Gross hematuria	Separate villous adenoma and non-muscle invasive bladder cancer	Synchronous urothelial carcinoma	TURBT followed by intravesical BCG
Qin et al. (2019) [16]	Urethral mass and urinary symptoms	Female urethral villous adenoma	Focal well-differentiated adenocarcinoma	Surgical excision; recurrence-free follow-up
Present case	Incidental asymptomatic detection	Diffuse plaque-like trigonal lesion extending across both vesicoureteric junctions	Cystitis cystica et glandularis with intestinal metaplasia; focal high-grade dysplasia; no invasive malignancy	Complete TURBT; recurrence-free during surveillance

Table 2 presents published reports and a case series of villous adenoma in the urinary system and highlights the distinctive clinicopathological features of this unusual condition [14-16]. Most reported cases originated from the bladder trigone, dome, urachus, or prostatic urethra [1,14-16]. These tumors are commonly associated with intestinal metaplasia or cystitis glandularis [1]. Importantly, several studies have documented synchronous or subsequent urothelial carcinoma and adenocarcinoma, emphasizing the malignant potential of the surrounding metaplastic field despite the benign histology of pure villous adenoma [15,16]. Most patients were successfully managed with complete surgical excision, including TURBT or partial cystectomy, with favorable outcomes in the absence of associated malignancy [14-16]. Compared with previously published reports, the present case is distinctive because of its entirely asymptomatic presentation and diffuse, plaque-like trigonal involvement extending across both vesicoureteric junctions, a clinicoradiologic pattern that has not been previously emphasized in the literature.

Our case is distinctive in demonstrating complete radiologic, endoscopic, and histopathologic correlation in an entirely asymptomatic patient with diffuse, plaque-like trigonal involvement. These findings further support the evidence for chronic intestinal metaplasia as a precursor environment for adenomatous transformation within the urinary bladder [1]. Despite the favorable postoperative outcome in the present case, periodic cystoscopic surveillance remains prudent because of the recognized association between villous adenoma and synchronous or subsequent malignancy [1,11].

Take-home messages

Villous adenoma of the urinary bladder is an exceptionally rare glandular neoplasm with significant diagnostic and clinical relevance because of its association with intestinal metaplasia [1] and potential coexistence with malignancy [1,11]. The present case is distinctive in demonstrating a completely asymptomatic, incidentally detected intestinal-type villous adenoma presenting as a diffuse, plaque-like trigonal lesion extending across both vesicoureteric junctions, an unusual clinicoradiologic pattern that has not been previously emphasized in the literature.

This case underscores the need for vigilance regarding the possibility of glandular neoplasms in unusual bladder lesions, particularly those presenting without symptoms. The strong correspondence among the imaging, endoscopic, and pathological findings in this case supports the irritation-metaplasia-adenoma model of bladder carcinogenesis [1]. The known association of villous adenoma with urothelial carcinoma or adenocarcinoma underscores the importance of careful evaluation and adequate tissue sampling to avoid missing an invasive tumor [1,15,16]. Complete resection followed by regular cystoscopic surveillance should be considered an important component of management, particularly in cases of intestinal metaplasia of the urothelium [1].

## Conclusions

Intestinal villous adenoma of the urinary bladder is a glandular tumor with intestinal differentiation that may occur in the setting of chronic irritation and metaplasia. The present case is distinctive in demonstrating an asymptomatic, plaque-like trigonal lesion with comprehensive radiologic, endoscopic, and histopathological correlation. Although villous adenomas are generally considered benign, their potential association with malignancy warrants thorough histopathological evaluation, complete transurethral resection, and ongoing surveillance.
